# Computational design of multi-epitope vaccine against Hepatitis C Virus infection using immunoinformatics techniques

**DOI:** 10.1371/journal.pone.0317520

**Published:** 2025-01-24

**Authors:** Sara Zubair, Fahed Parvaiz, Turki Abualait, Khalid Al-Regaiey, Tasneem Anwar, Mahnoor Zafar, Imdad Kaleem, Shahid Bashir

**Affiliations:** 1 Department of Biosciences, COMSATS University Islamabad (CUI), Islamabad, Pakistan; 2 College of Applied Medical Sciences, Imam Abdulrahman Bin Faisal University, Dammam, Saudi Arabia; 3 Department of Physiology, King Saud University, Riyadh, Saudi Arabia; 4 Neuroscience Center, King Fahad Specialist Hospital Dammam, Dammam, Saudi Arabia; Cholistan University of Veterinary and Animal Sciences, PAKISTAN

## Abstract

Hepatitis C Virus (HCV) is a blood borne pathogen that affects around 200 million individuals worldwide. Immunizations against the Hepatitis C Virus are intended to enhance T-cell responses and have been identified as a crucial component of successful antiviral therapy. Nevertheless, attempts to mediate clinically relevant anti-HCV activity in people have mainly failed, despite the vaccines present satisfactory progress. In this study, we used an array of immunoinformatics approaches to design a multiepitope peptide-based vaccine against HCV by emphasizing 6 conserved epitopes from viral protein NS5B. The potential epitopes were examined for their possible antigenic combination with each other along with GPGPG linkers using structural modeling and epitope-epitope interaction analysis. An adjuvant (β-defensin) was introduced to the N-terminus to increase the immunogenicity of the vaccine construct. Molecular dynamics simulation discloses the most stable structure of the proposed vaccine. The designed vaccine is potentially antigenic in nature and can form stable and significant interaction with both receptors TLR2 and TLR3. The vaccine construct was also subjected to *In-Silico* cloning which confirmed its expression efficiency in a vector. The findings indicate that the designed multi-epitope vaccine have a great potential for preclinical and clinical research, which is an important step in addressing the problems related to HCV infection.

## Introduction

In 1989, the primary cause of non-A, non-B hepatitis was identified as HCV, a global blood-borne virus that causes severe infection of the liver.

Around 200 million HCV cases are reported across the globe, and up to five million new cases are diagnosed each year. In Pakistan, HCV infection rate is the second highest in the world after Egypt with one in every 20 people that are being affected. Individuals suffering from chronic HCV infection usually remain untreated or undiagnosed and eventually lead towards liver cancer, cirrhosis, and severe fibrosis [1]. HCV is likely to be transmitted through blood and blood borne products. Nevertheless, HCV can also be transmitted from mother to fetus. Several risk factors such as immunological response, jaundice like symptoms, viral genotype and sub-types, and ethnic group, influence the chronic HCV infection.

HCV can travel through the bloodstream as a lipo-viral particle, which is made up of lipoproteins that are firmly attached to the HCV particle. Among all these HCV proteins, the serine proteases (NS3-4A) and the RNA-dependent RNA polymerases (NS5B-RdRp) are thought to be the primary focus of anti-HCV medication development. Owing to the high genetic diversity of HCV, there are seven primary genotypes and more than 60 subtypes. The difference at nucleotide level is 30% between genotype and the 15% subtype. Furthermore, HCV exhibits a tremendous amount of genetic variation within an infected person, where it persists as a quasi-species brought on by both the high viral replication rate and the elevated mistake rate of HCV polymerase.

Due to high prevalence worldwide, the disease burden, and conventional methods of transmission draw attention towards the diagnosis, treatment, and prevention of HCV. The development of recently developed direct-acting antivirals (DAAs), which have significantly altered the HCV landscape in recent years, may cure more than 95% of those with HCV infection [2]. Despite the high sustained virological response (SVR) due to use >90% of DAA, they may not lower the risk of HCC due to HCV infection. Secondly, drug resistance arises in patients using DAA due to mutation in HCV variants [3]. Thirdly, DAA are expensive and inaccessible in most developed countries. These factors contribute towards highlighting the potential need of designing a vaccine candidate against HCV infection.

Several HCV vaccine candidates have been studied in vivo as well as in clinical trials, however, no HCV vaccine has been commercially available till now. The high rate of virus mutation, and deliberate avoidance of B- and T-cell reactions, is one of obstacles to the development of an efficient vaccine against HCV. The variation in virus genetics, which has seven main genotypes and numerous subgroups, is also a significant obstacle in the vaccine development. So, for that purpose designing a multiepitope vaccine with great conservatism against HCV using computational vaccinology methods is a potential solution to this issue. In addition, the In-silico designed vaccines poses immunogenic markers and other parameters, such as the restricted epitopes of major histocompatibility complexes (MHC)-I and MHC-II that T-cell clones target, multiepitope vaccines are preferable to conventional vaccines. The methods of computational vaccinology have been chosen to develop a novel multiepitope peptide vaccine against HCV.

In this study, we develop a vaccine based on multiple epitopes by using *In-silico* approach against HCV. Compared to conventional empirical methodologies, this multidisciplinary approach has several benefits. By reducing the need for time-consuming experimental testing and increasing the accuracy of the design process, it speeds up the identification and evaluation of viable vaccine candidates. Additionally, it permits the customization of vaccine components to certain population groups based on genetic and epidemiological data, a crucial component for developing effective immunization strategies for HCV.

With millions of infected individuals globally, HCV continues to be a major cause of chronic liver disease. The lack of a vaccine and awareness among people highlights the necessity of preventive measures as well as development of the effective vaccination against HCV infection.

## Material and method

**Fig 1:**
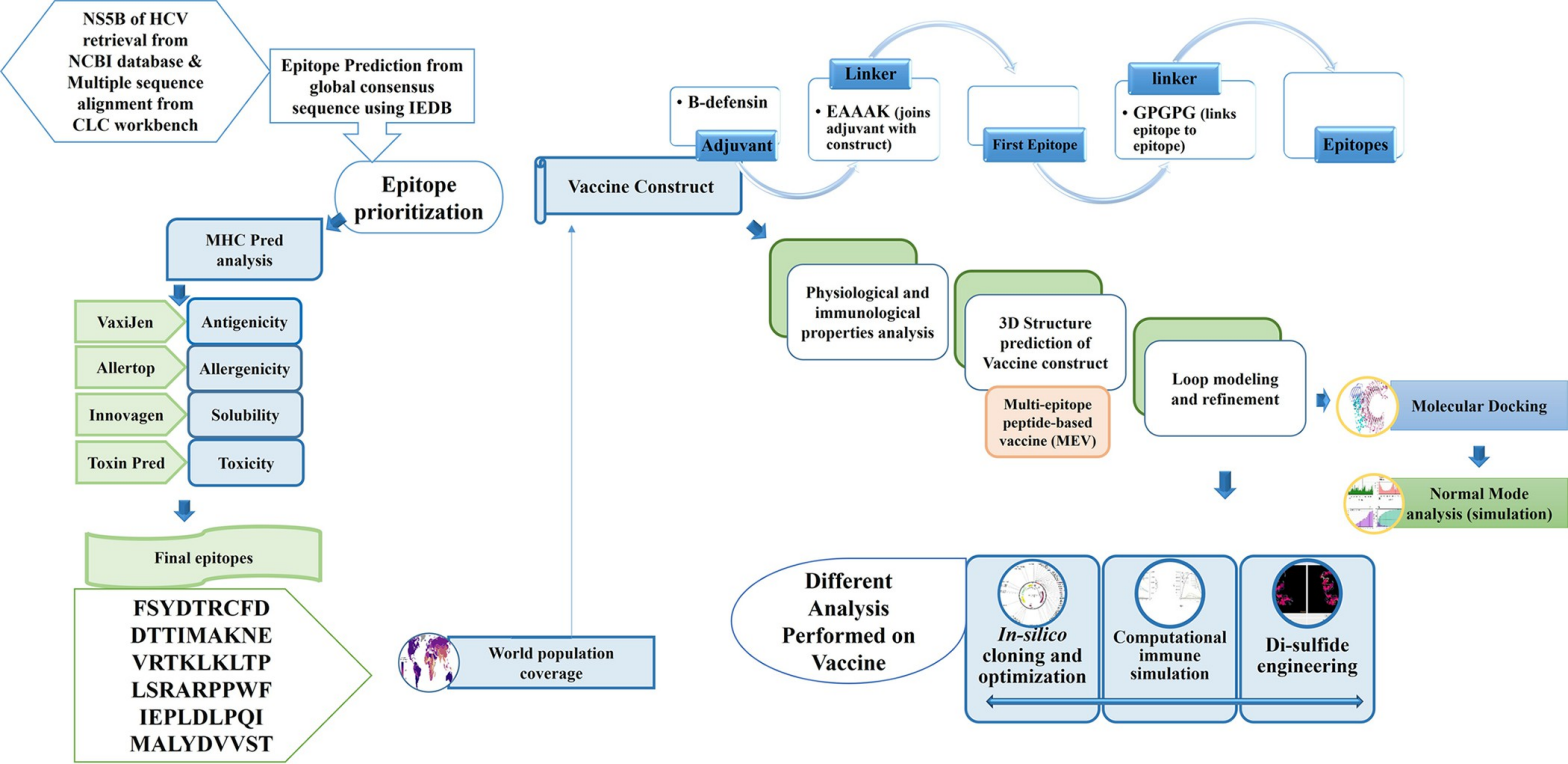
Schematic representation of steps followed throughout the construction of multiepitope vaccine construct.

The complete research methodology used to design a multiepitope vaccine against HCV is explained in Fig 1.

### 1. Targeted epitope prediction

The National Center for Biotechnology Information database (NCBI) (https://www.ncbi.nlm.nih.gov) was used to extract the protein sequence of the NS5B protein, which is part of the polyprotein sequence of the HCV (https://www.ncbi.nlm.nih.gov).Multiple sequence alignment of NS5B protein of HCV were performed using CLC workbench to obtain the global consensus sequence.CLC Workbench is made to effectively process, analyze, and display biological data, including protein structures and genetic sequences. These methods include phylogenetic tree construction, variant calling (with tools like GATK), multiple sequence alignment, expression data analysis, and sequence alignment (e.g., BLAST, ClustalW). Additionally, CLC Workbench uses machine learning methods for data integration and predictive modeling, which enables users to find intricate patterns in genomic data. The software is a complete tool for molecular biology and genetics researchers since it also includes a number of statistical [4]. It was also used for subsequent epitope prediction (https://bio.tools/abcpred) Utilizing its optimization capabilities, the Artificial Bee Colony (ABC) algorithm has been modified for B-cell epitope prediction in order to find putative antigenic determinants that are essential for immunotherapy and vaccine development. New developments use hybrid and machine learning techniques to increase the precision of linear and conformational B-cell epitope predictions. For instance, ABC-based techniques improve the performance of classifiers like support vector machines or neural networks by optimizing the selection of characteristics from antigen sequences, such as hydrophobicity and solvent accessibility. With encouraging results for both linear and 3D conformational epitopes, these methods have been applied to a variety of datasets, supporting the logical development of vaccines against infectious agents [5]. To find epitopes that CD8+ and CD4+ T- lymphocytes recognize, the Immune Epitope Database (IEDB) tools employ algorithms that make use of MHC-binding predictions. To predict epitope binding affinity to different HLA alleles, one well-known tool, TepiTool, uses state-of-the-art techniques like NetMHCpan, guaranteeing wide population coverage. To improve prediction accuracy, recent developments in IEDB algorithms have combined immunopeptidomics with extensive datasets [6](http://tools.iedb.org/main/tcell/) [7]. The capacity of epitopes to be non-allergenic, non-toxic, anti-inflammatory, and non-homologous to human proteins, in addition to being sufficiently antigenic to trigger an immune response, are the main requirements for acceptance as a potentially effective vaccine design. Increased T- cell coverage in the population may be a sign of a potential vaccine. VaxiJen (http://www.ddg-pharmfac.net/vaxijen/VaxiJen/VaxiJen.html) was used to assess the antigenicity of epitopes. A web-based program called the VaxiJen algorithm was created specifically for vaccine design and antigenicity prediction. It employs a machine learning-based method that relies on the physicochemical characteristics of the sequences rather than sequence alignment to categorize proteins as antigens or non-antigens. To create peptide-based vaccines, immunoinformatics workflows have successfully employed VaxiJen [8,9].Accuracy of VaxiJen is almost 88% [10] allergenicity using AllerTop (https://www.ddg-pharmfac.net/AllerTOP/method.html) A bioinformatics technique called the AllerTOP algorithm was created to forecast a protein’s allergenicity by examining its physicochemical characteristics. It uses machine learning techniques, specifically the k-nearest neighbors (kNN) algorithm, to categorize proteins as allergens or non-allergens by converting protein sequences into vectors using auto- and cross-covariance transformations. High sensitivity (94%) has been demonstrated by AllerTOP, which is especially useful for anticipating the allergen exposure pathway (food, inhalant, or toxin, for example) [11]. Solubility testing was performed using INNOVAGEN (http://www.innovagen.com/). The strategy used by Innovagen, which is represented in their PeptideCADTM program, optimizes peptide solubility, surface exposure, and antigenicity to design peptides for antibody manufacturing. By forecasting sequence characteristics essential for antigen binding and immune response, this algorithm helps to choose immunogenic peptides, a process essential to the production of vaccines and therapeutic antibodies. A computational technique called the ToxinPred algorithm is used to forecast the toxicity of proteins and peptides. The recent version of ToxinPred 3.0, has a high AUROC of 0.98 and an MCC of 0.81 for toxicity prediction [12](https://webs.iiitd.edu.in/raghava/toxinpred/)) [13].The most promising epitopes were chosen and their combinations were made with the right adjuvants and linkers to create the multi-epitope vaccine. GPGPG and EAAAK linkers, crucial for providing flexibility, folding, stability, and functional domain separation, were used to adjoin epitopes produced from various immunoinformatic programs to create the final vaccine.

When used in the vaccine design, linkers like GPGPG and EAAAK have been shown to enhance the immunogenicity, stability, and immune response of multi-epitope vaccines. By promoting appropriate folding of epitopes, the GPGPG linker improves their presentation and is essential for effective immune system recognition. On the other hand, the EAAAK linker greatly stabilizes the protein structure, ensuring that the vaccine remains intact and effective during processing and within the host organism. As a result, these linkers help to develop vaccines that are both effective and able to trigger a potent immune response [14].

The final vaccine design aimed to enhance immunogenicity and facilitate epitope presentation to the immune system. Using the ProtParam Tool on the Expassy server (https://web.expasy.org/protparam/). The Swiss Bioinformatics Resource Portal’s (ExPASy) ProtParam method computes chemical and physical characteristics of a protein sequence. These comprise, among other things, the estimated half-life, extinction coefficient, instability index, molecular weight, isoelectric point (pI), and amino acid composition EXPASY. The program is frequently used to evaluate protein sequences for critical characteristics including hydropathy (using GRAVY) and stability (using the instability index), as well as to forecast characteristics [15]. The physiochemical characteristics of the Multiepitope Peptide Vaccine Construct were examined. Physiochemical properties provide important details on the vaccine’s molecular makeup and structural characteristics, which are crucial to both its effectiveness and suitability as a vaccine candidate. Its population coverage was checked using the Population coverage of IEDB (http://tools.iedb.org/population/result/). The IEDB (Immune Epitope Database) Population Coverage algorithm calculates the proportion of individuals in a population likely to respond to specific epitopes based on HLA allele frequencies and MHC binding data. It integrates predicted MHC class I and class II binding affinities with the genetic variability in human populations to estimate how well a set of epitopes covers diverse global populations [16].Numerous other attributes were also investigated, such as the molecular weight, stability index, and GRAVY value [17].

### 2. Prediction of discontinuous B-cell epitope

To predict discontinuous epitopes from the vaccine’s 3D structure, an ellipro structure-based technique was employed. This tool clusters discontinuous epitopes using the distance between the residue’s center of mass and PI (protrusion index) values. The prediction’s outcome reveals Conformational B-cell epitopes with a range score of 0.74 to 0.52 and consisting of 75 residues.

The ElliPro method uses protein structural analysis to predict antibody epitopes. It finds areas that are likely to interact with antibodies by using geometric features, especially the protrusion of residues from the protein surface. This technique works well for predicting discontinuous epitopes from 3D structures and doesn’t require training data [18].

### 3. Multi-epitope based vaccine formulation

A multi-epitope-based vaccine can elicit a robust immune response and initiate adaptive immunity. The final epitopes that were chosen were joined together using linker GPGPG. To increase the potential of the vaccine we linked adjuvant B-defensin with linker EAAAK [19]. Afterward, the SCRATCH protein predictor (https://scratch.proteomics.ics.uci.edu/) was used to model the vaccine tertiary structure [20]. A range of algorithms are used by the SCRATCH protein predictor to forecast protein characteristics and structure, including secondary structure, solvent accessibility, and disordered regions. To obtain precise findings in structure prediction, these predictors use evolutionary data, structural templates, and machine learning models, such as support vector machines (SVMs) [21].

USCF chimera was used to analyze the 3D structure of the vaccine construct. A range of algorithms are used by the UCSF Chimera software, mainly for data representation, structural analysis, and molecular visualization. It has excellent performance and usefulness in manipulating density maps, analyzing molecular interactions, and visualizing 3D molecular structures. Additionally, it connects with molecular docking tools such as AutoDock Vina for protein-ligand interaction prediction [22].

A peptide-based vaccine construct’s 3D structure was refined using the galaxy web (https://galaxy.seoklab.org/). Then PDB Sum was used to predict Ramachandran plots for the designed vaccines. Comprehensive, visual summaries of 3D protein structures that have been stored in the Protein Data Bank (PDB) are produced using the PDBsum program. Along with providing information on secondary structural features and functional sites, it offers schematic representations that illustrate molecular interactions, such as those involving protein chains, ligands, and metal ions [23].

### 4. Disulfide engineering and expression analysis

Disulfide engineering was used to get rid of any potential structural instability and improve the vaccine’s overall stability. Disulfide linkages are created between cysteine residues in the protein sequence using a process known as Disulfide engineering (http://cptweb.cpt.wayne.edu/DbD2/index.php) [24]. Protein disulfide bonds are predicted and engineered using the Disulfide by Design (DbD2) algorithm, which examines structural and geometric properties with an emphasis on the torsion angles of cysteine residues. It evaluates the viability of the suggested disulfide bridge using energy functions derived from reported distributions of native disulfide bonds in protein structures [25].

To maximize codon utilization for gene expression in a variety of hosts, especially *E*. *coli*, the Java Codon Adaptation Tool (JCat) algorithm was created. It increases the effectiveness of heterologous protein production by matching the codons in a target gene with those commonly utilized in the expression system. This approach eliminates the need for manual codon table curation as its quick and easy to utilize [26].

Codon optimal expression is done to improve optimal expression, replacing rare or suboptimal codons in the DNA, and lower the chance of potential translational mistakes, which can also affect vaccine immunogenicity and stability therefore to calculate the levels of protein expression, Using the Java Codon Adaptation Tool (JCat) service, the codon adaptation index (CAI) values and GC contents of the *E*. *coli* (strain K12) codon system were determined. The GC content ranges from 30% to 70%, and a score of > 0.8 is regarded as desirable. The optimal CAI score is 1.0.

A program called SnapGene is used to make molecular biology tasks like plasmid production, primer creation, and DNA sequence annotation more efficient. It contains algorithms to mimic cloning processes, PCR, and assembly techniques like Gibson and Golden Gate AR5IV SNAPGENE, and it uses a graphical user interface (GUI) to visualize DNA operations.The program helps researchers effectively organize and visualize cloning studies by supporting a variety of techniques for sequence alignment and feature annotation [27]. To enable effective protein expression, the final vaccine construct was inserted into the expression vector pET-28a(+) using SnapGene [28].

### 5. Computational immune simulation

The C-IMMSim (Clustering-based Immune Multi-Agent Simulation) algorithm solves complex optimization problems. It specifically makes use of the concepts of clustering and multi-agent systems to efficiently explore solution spaces. The approach combines clustering techniques with immune-based strategies, like memory cells and antibody-based matching, to improve optimization performance in a variety of fields [29]. By simulating the behavior of the immune system, (https://kraken.iac.rm.cnr.it/C-IMMSIM/index.php), C-IMMSim was employed to evaluate the vaccine’s immunogenicity and predict potential immune responses. This simulation provides crucial insights into the vaccine’s ability to activate the immune system.

### 6. Molecular docking between vaccine construct and immune receptor

The interplay between immune cells and the vaccine is necessary for the development of a stable immune response. Molecular docking was conducted using clusPro2.0 (https://cluspro.org/login.php) to assess the epitopes affinity for binding with the TLR receptors. ClusPro is a protein-protein docking method that creates a number of possible docking poses and then clusters them to choose the best configuration among protein interactions. The binding space is explored using Monte Carlo simulations, and the final docking model selection is based on energy-based criteria [30]

### 7. Molecular docking of the epitopes binding to HLA-A1

For molecular docking to be performed, the 3D structure of specific epitopes binding to HLA-A1 was employed. The 3D structure of certain epitopes was created using Cluspro, which uses the input to predict the peptide’s tertiary structure.

### 8. Normal mode analysis of vaccine construct

Using the online program iMODS (https://bio.tools/imods) , normal mode analysis was performed to explain the typical protein motion within intrinsic coordinates. Using this tool, the deformation of the structure, the RMSD values, the covariance between individual residues, the Eigenvalue of interacted residues, and the rest were investigated. By carefully examining the coordinates, it ascertains the stability of the complexes. Using a differential evolution framework and various mutation techniques, the I-MODE algorithm (Improved Multi-Operator Differential Evolution) is intended to handle complicated optimization problems [31].

## Results and discussion

### 1. Evaluation of targeted epitopes

**Fig 2:**
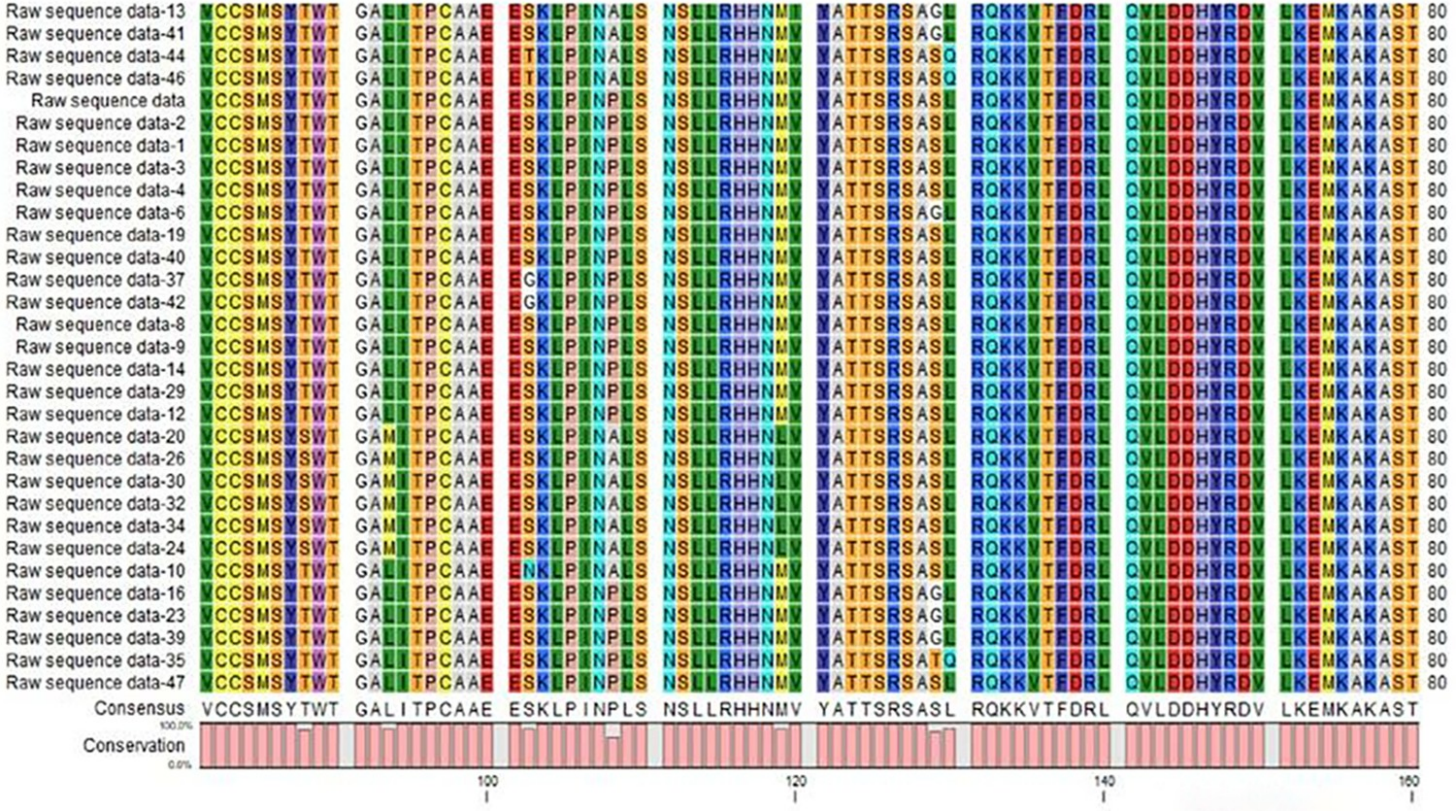
Multiple sequence alignment and global consensus sequence.

We design multi-epitope peptide-based vaccine against HCV, which targets NS5B, RNA-dependent RNA polymerase, of HCV. The consensus sequence provides valuable insights into the conserved regions within the HCV protein, which are crucial to design a potential. The global consensus sequence in (Fig 2) will serve as the basis for predicting B-cell epitopes and subsequently identifying potential MHC-II and MHC-I epitopes ABC pred and IEBD, an immune epitope-based database tool. The results of antigenicity indicated that every targeted peptide sequence was antigenic by using VaxiJen 2.0 server (threshold 0.6). The allergenicity of the anticipated epitopes were predicted using Aller Top 2.0. By targeting conserved regions in the viral protein, we aim to elicit a broad and robust immune response that can confer protection against various HCV strains. Non-toxic and good water-soluble epitopes were used for the designing of multi-epitope vaccine construct. Six promising epitopes were selected which include **FSYDTRCFD, DTTI MAKNE, LSRARPRWF, IEPLDLPQI, VRTKLKLTP**, and **MALYDVVST (Table 1)**. These epitopes were thought to be excellent prospects for stimulating powerful and targeted immune responses against HCV in the human body.

**Table 1:** Shortlisted potential epitopes after prioritization.

Epitopes	Antigenicity	Antigenic score	Toxicity	Allergenicity	Solubility
FYSDTRCFD	antigenic	1.55	Non -toxic	Non allergen	Good water soluble
DTTIMAKNE	antigenic	0.89	Non -toxic	Non allergen	Good water soluble
LSRAPRWF	antigenic	0.96	Non -toxic	Non allergen	Good water soluble
IEPLDLPQI	antigenic	1.23	Non -toxic	Non allergen	Good water soluble
VRTKLKLTP	antigenic	2.19	Non -toxic	Non allergen	Good water soluble
MALYDVVST	antigenic	0.68	Non -toxic	Non allergen	Good water soluble

### 2. Screening of discontinuous B-cell epitope

Protein folding into its natural three-dimensional shape is necessary for discontinuous B-cell epitope formation. The immune system’s ability to recognize discontinuous epitopes is essential, however, the amino acids that make up these epitopes may be widely apart in the main sequence, yet they come together in the folded structure of proteins to form the epitope.

Using the IEBD ellipro server, discontinuous B-cell epitopes for the multi-epitope vaccination were predicted. The prediction parameters (minimum residue score and maximum distance) were set to their default values when the refined protein was uploaded in PDB format. Ellipro predicted varying numbers of epitopes in each protein based on the threshold values for parameters R and S; for example, given a R of 6Å and S of 0.5 (Ellipro: a new structure-based tool for the prediction of antibody epitopes)

**Fig 3:**
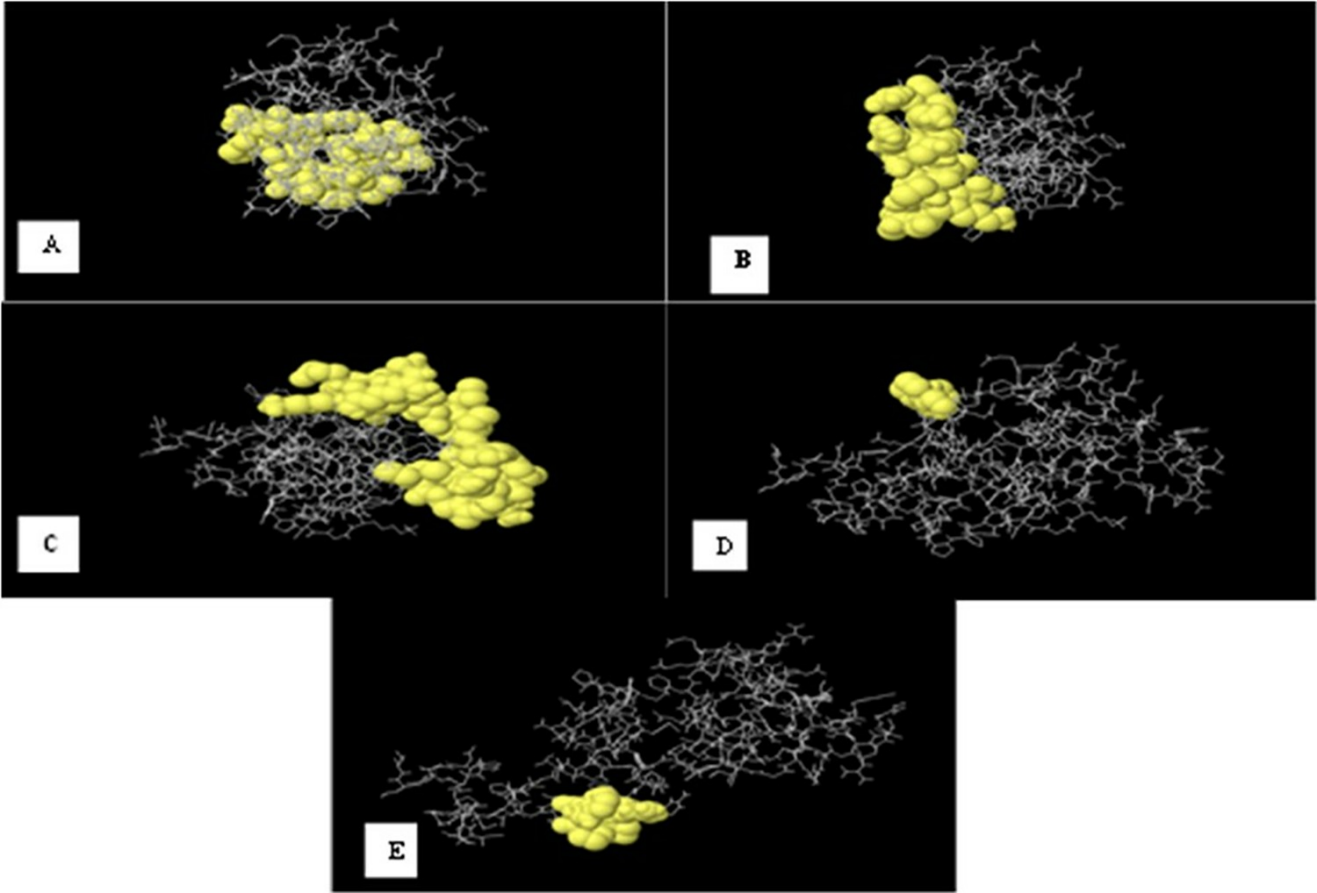
The 3D model of 5 predicted conformational epitopes. The yellow regions are the conformational B-cell epitopes, while the grey regions are the residue remnant. (A) 19 residues with 0.749 score. (B) 11 residues with 0.703 score. (C) 33 residues with 0.623 score. (D) 6 residues with 0.614 score. (E) 6 residues with 0.525 score.

Ellipro predicted five epitopes with score of 0.792, 0.726, 0.672, 0.636, 0.521 and visualization of predicted epitopes by J/mol where epitopes are shown in yellow color and rest of protein in violet color in Fig 3. Ellipro predicted varying numbers of epitopes in each protein based on the threshold values for parameters R and S; for example, given a R of 6Å and S of 0.5

### 3. Population coverage analysis

**Fig 4:**
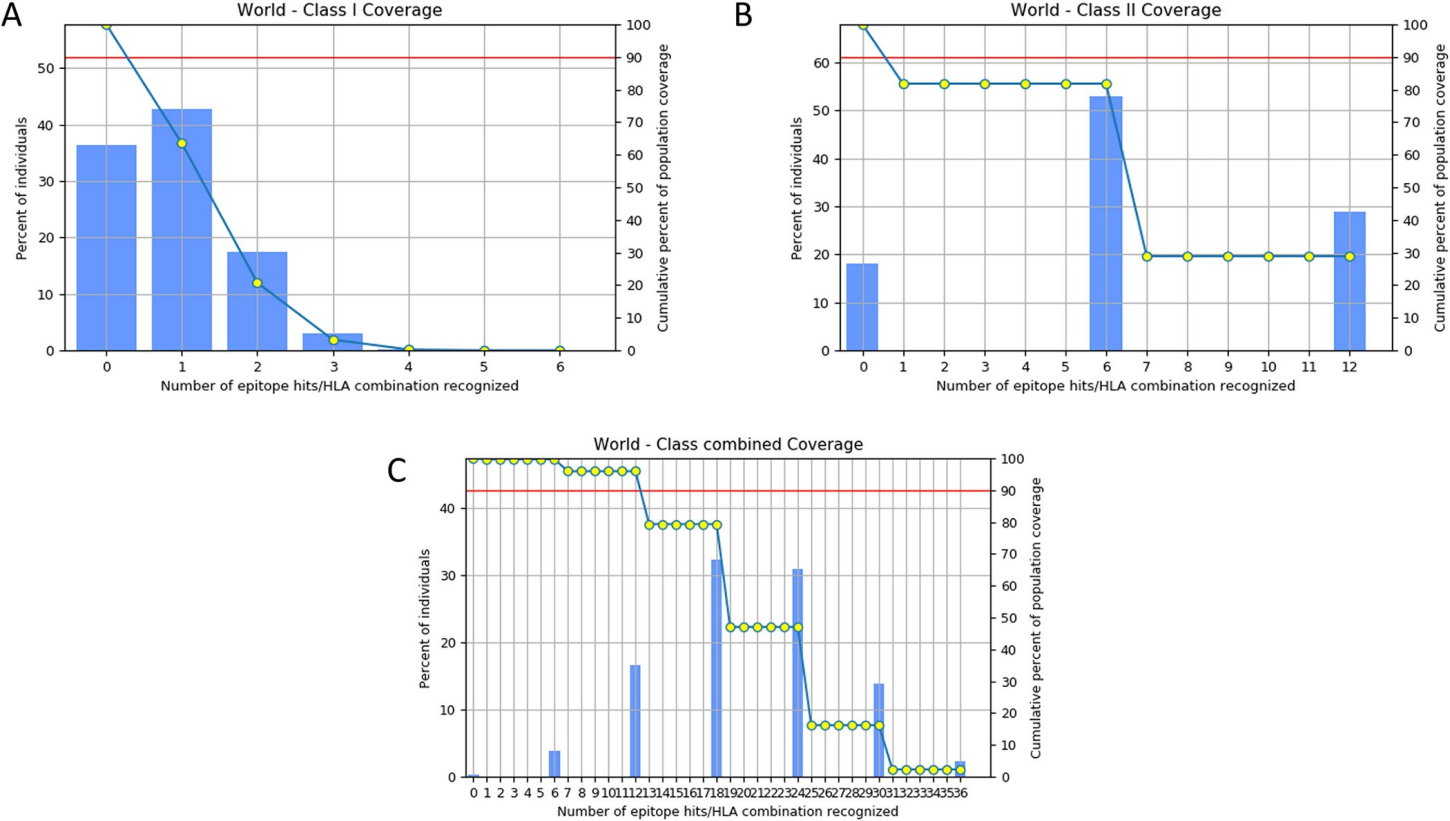
(A) Represents the world coverage of MHC I which is 98.55%. (B) Represent the world coverage of MHC II which is 81.81%. (C) Represent the combined MHC class coverage of 99.74%.

Population coverage analysis was conducted to assess the possible effectiveness and coverage of the planned multi-epitopes vaccine against HCV. An examination of population coverage calculates the proportion of the world’s population that carries human leukocyte antigen (HLA) alleles that can successfully introduce the vaccine’s epitopes to the immune system. As a result, it might also affect the creation of efficient epitope-based vaccine[32]. It is possible to estimate the built vaccine epitope coverage in the target population using the IEDB population coverage tool. This tool reveals information on how different populations worldwide differ in terms of HLA allele distribution. Ethnicity and location affect the prevalence and expression of certain HLA alleles. Then six potential epitopes were used as an input to forecast the probable HLA allele coverage. According to the results of the population coverage analysis, a sizable percentage of people worldwide are protected by the multi-epitope vaccine that was developed. Its overall coverage was 98.55% Fig 4A for MHC I and 81.81% Fig 4B coverage for MHC II and the combined coverage was 99.74% Fig 4C.

### 4. Vaccine construct designing and Physiochemical analysis

**Fig 5:**
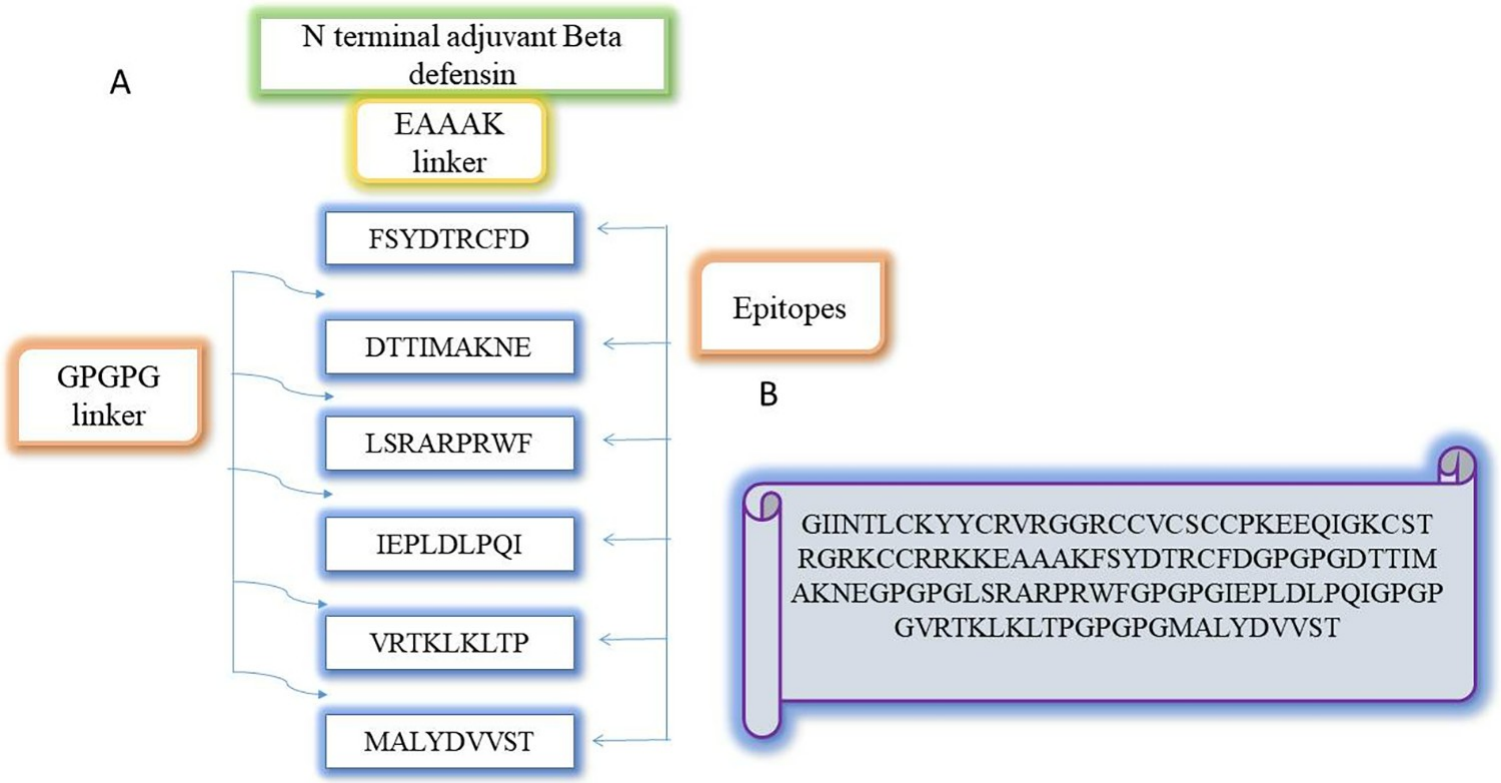
Schematic representation of the final multi-epitope vaccine constructs. The 129-amino acid long peptide contains β-defensin as an adjuvant at the N-terminal followed by epitopes along with linkers.

The vaccine construct was linked to β-defensin using the EAAAK linker to boost immunogenicity and improve the immune response against the antigen as shown in the **Fig 5**. GPGPG connects each epitope, allowing for the creation of a multiple epitope vaccine construct. Furthermore, we performed an extensive physiochemical properties analysis to make sure the developed multi-epitope vaccine against HCV is stable, bioactive, and safe. This research offers vital details on the vaccine’s molecular makeup and structural characteristics, which are crucial to both its effectiveness and suitability as a vaccine candidate. For the examination of the physiochemical properties, the Expasy ProtParam tool was used. The physiochemical properties analyzed depict that construct contains 129 amino acids with a molecular weight of 13.7 **kda** and theoretical pI of 9.43. The computed value of the instability index (II) is **36.75**. The protein is therefore categorized as stable. **51.19** m is the aliphatic index. A -**0.501** GRAVY value is negative which shows it signifying hydrophilic nature half-life in mammalian reticulocytes, invitro is 30 hours in Escherichia coli in vivo >10 hours and in yeast in vivo >20 hours as shown in **Table 2**.

**Table 2:** Physiochemical properties of the designed multi-epitope vaccine.

No of amino acid	129
Molecular weight	13.7kDa
Theoretical index	9.43
Estimated half life (mammalian reticulocytes, invitro) (Escherichia coli ,in vivo) (yeast in vivo)	30 hours >10 hours >20 hours
Instability index	36.75
Aliphatic index	55.19
Grand average	-0.501

The vaccine’s general structure and stability was clarified by the investigation of its physiochemical properties. A good molecular weight, a theoretical pI, and an effective extinction coefficient were all displayed by the vaccine. A low instability score for the vaccine also suggested high structural stability and the possibility of a longer shelf-life when stored. The vaccine was anticipated to have strong thermal stability, which is essential for storage and transit under a variety of environmental circumstances, according to the aliphatic index.

### 5. Loop modeling and refinement

The tertiary structure of the vaccine’s loops was modelled using the Galaxy Loop tool of GalaxyWeb to ensure appropriate structural representation. Loops, which are frequently flexible sections in protein structures, can be improved in terms of conformation and shape by loop modeling. The Galaxy Refine tool was then used to refine the overall structure [33]. The process of refining improves the structural accuracy and stability of the vaccine’s tertiary structure by optimizing its overall shape and energy [34].

### 6. Secondary, tertiary structure prediction and ramachandran plot

**Fig 6:**
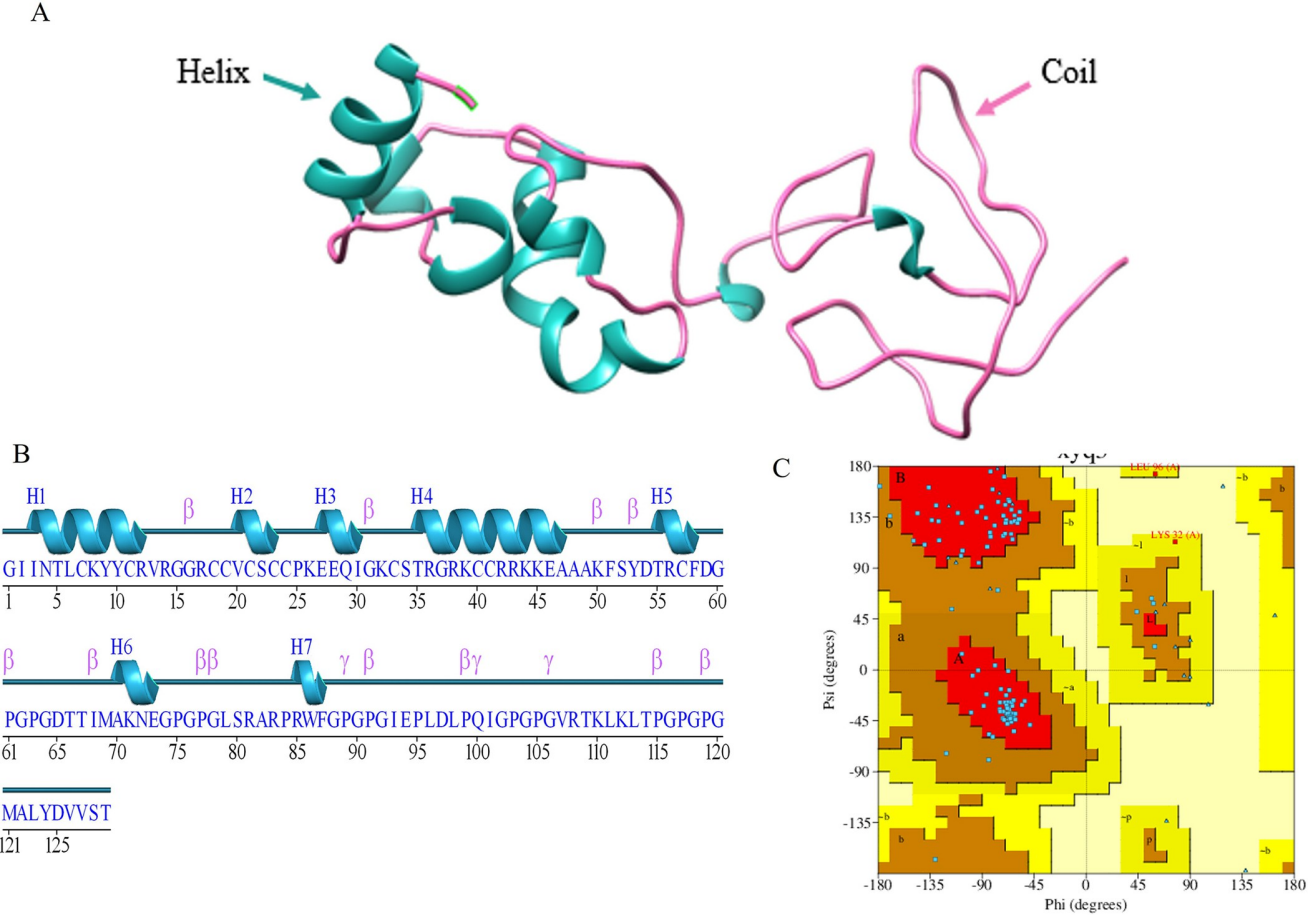
Tertiary structure model of multi-epitope vaccine construction and validation. (A) 3D vaccine construct showing coil and helix (B) Secondary structure for multi-epitope vaccine construct (C) Ramachandran plot for the multi-epitope vaccine construct.

The 3D pro of scratch protein predictor was utilized to predict the tertiary structure of the proposed multi-epitope HCV vaccine (Fig 6A) that generates three-dimensional protein structures from existing protein structures with related sequences using homology-based modeling techniques. The vaccine construct’s secondary structure revealed that it had 7 helices, 7 helix-helix interactions, 12 beta turns, and 3 gamma turns (Fig 6B).

The flexibility and structural dynamics of the vaccine construct are reflected in the 3D structure shown in Fig 6, which is primarily composed of random coils with seven brief helical sections. While helical sections add to structural stability and possible receptor-binding surfaces, coils frequently signal regions of flexibility that are essential for antigen processing and presentation. For effective interaction with immune system components like T-cell receptors or antibodies, this flexibility/rigidity balance is crucial. The quality of the model should be thoroughly assessed utilizing tools such as Ramachandran plots for stereochemical evaluation. Validation guarantees that the design may effectively elicit an immunological response and is structurally feasible. To confirm the model’s usefulness, specifics regarding these instruments and their assessment metrics such as residue geometry and error rates are essential.

In this study, pdbsum structural database was used to generate protein 3D structure and to assess the quality of tertiary structure [35]. The Ramachandran plot examines the protein’s backbone dihedral angles (phi and psi) and identifies any places that are sterically permissible or prohibited. The results indicated that 87.1% of the structure is under the favorable region, 2.2% is under the allowed region, and 0% is under the disallowed region as shown in Fig 6C.

### 7. Codon optimization and *in silico* cloning

To maximize the codon utilization of the vaccine design, the Java Codon Adaptation Tool (JCat) was utilized in *E*. *coli* (strain K12) for maximum protein production. The adapted sequence average GC content was 64.4.2%, indicating a high expression in the *E*. *coli* host, and the codon optimization index (CAI) value was predicted to be 1.0. Codon optimization also lowers the chance of potential translational mistakes, such as premature termination or misfolding, which can affect the vaccine’s immunogenicity and stability. The optimized DNA sequence obtained from JCat was then used for the subsequent steps. This codon-optimized DNA sequence is vital for obtaining high yields of the multi-epitope vaccine during the protein expression process.

**Fig 7:**
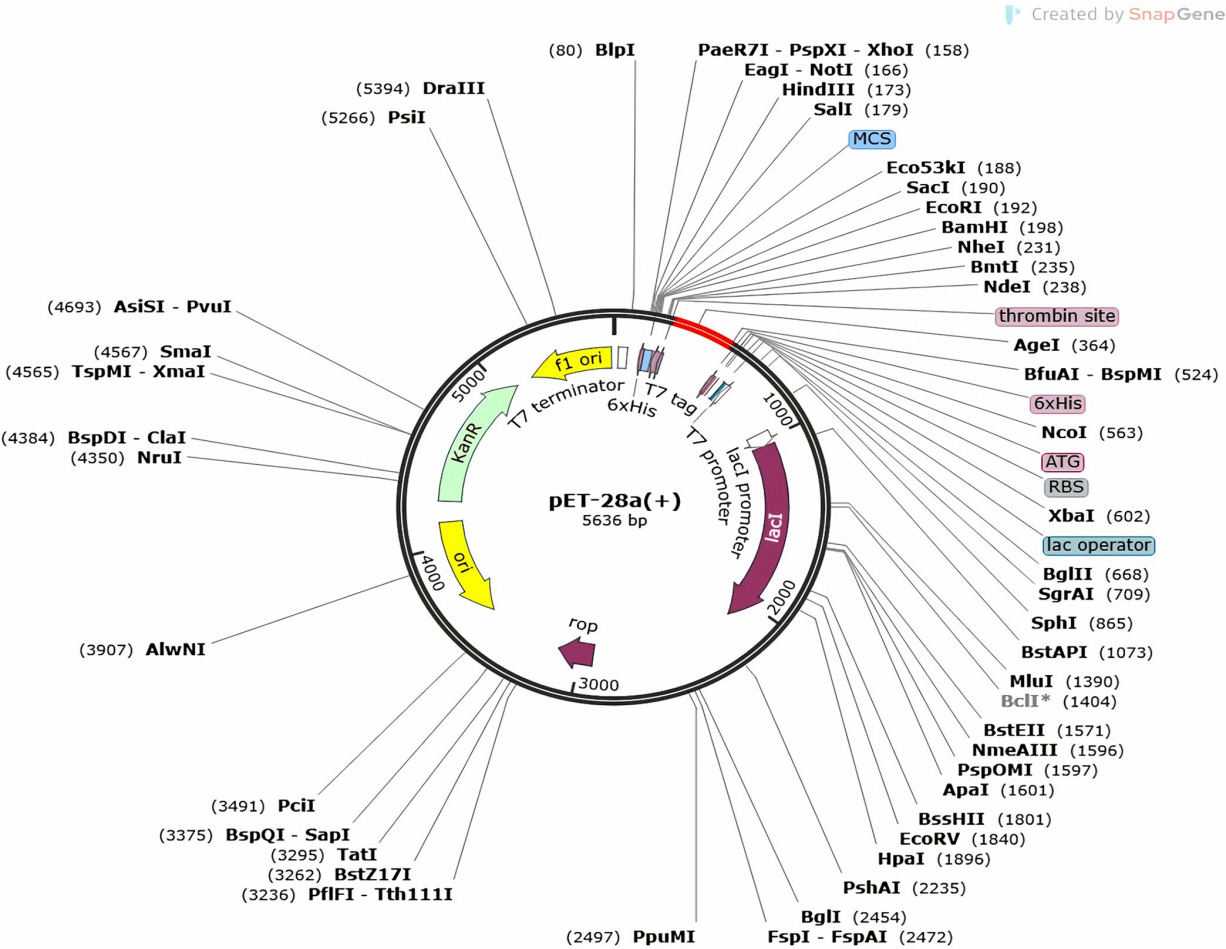
*In-silico* restriction cloning of the gene sequence of the vaccine construct into the pET28a(+) expression vector. The red part indicates the gene coding the multi-epitope vaccine construct, and the black part indicates the vector backbone.

Using the SnapGene tool, the optimized structure of multi-epitopes vaccine was added to the expression vector pET-28a(+). The *In-silico* cloning process ensures that the genetic sequence is integrated correctly into the vaccine into the vector for effective protein expression (as shown in Fig 7). In this way, a prokaryote like Escherichia coli (*E*. *coli*) may produce huge amounts of the vaccine [36].

### 8. Disulfide engineering

**Fig 8:**
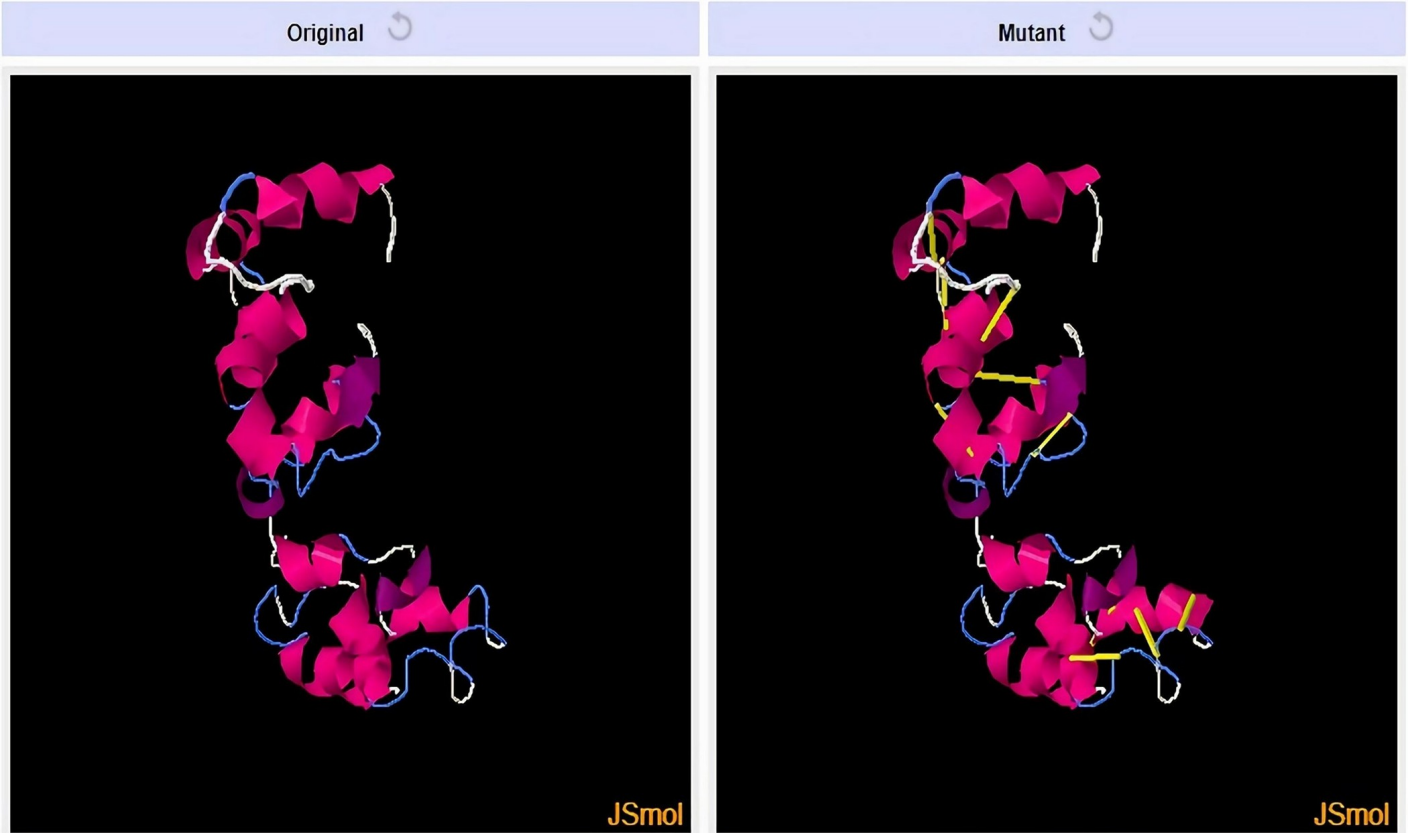
Mutant model for vaccine generated by introducing cysteine residues (shown in yellow).

In the disulfide engineering process, we locate appropriate positions in the vaccine’s structure to insert a disulfide bond in it. These ties were thoughtfully positioned to strengthen designated areas and reduce structural instability. The multi-epitope vaccine tertiary structure is strengthened using disulfide engineering, increasing its capacity to endure environmental stress and preserving conformational stability throughout storage and transportation. Fig 8 contains the original and the mutant model for the vaccine. The amino acid pairs mutated as cysteine are given in Table 3.

**Table 3:** Residue pairs mutated as cysteine (Disulfide engineering).

Residue 1	Residue 2	χ_3_	kcal/mol
21 CYS	57 CYS	-93.66	1.91
18 CYS	23 CYS	-76.16	3.12
77 PRO	99 PRO	-88.16	4.03

### 9. Computational immune simulation

**Fig 9:**
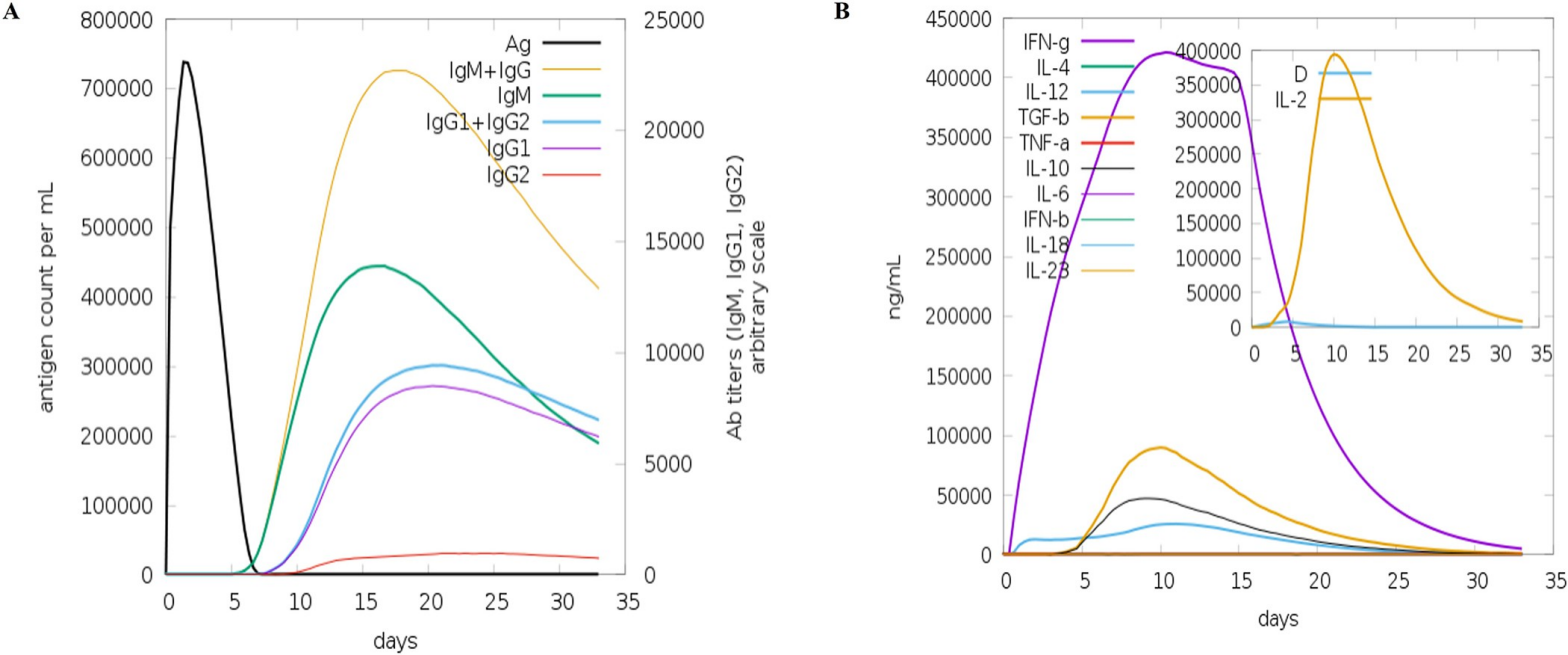
(A) Antigen level increase from day 0 to day 5 shown by the black line in the above-given graph. Antibody IgM+IgG level increased from day 5 shown in the yellow color line. Then IgG1+IgG2 is shown by a green line. (B) Concentration of cytokines and interleukins. The inset plot shows a danger signal together with leukocyte growth factor IL-2.

ImmSim server was used to simulate the immune profiles of the HCV vaccine. The immune response to the chimeric vaccine was comparable with actual immunological responses, with stronger tertiary and secondary responses. The C-ImmSim server simulates the thymus, lymph nodes, and bone marrow, three physiological organs [33]. Immunity to vaccine antigens was significantly influenced by all primary, secondary, and tertiary immune responses. Furthermore, IgM ^+^ IgG was seen in the higher titers, which was followed by IgG1 ^+^ IgG2 and IgM as shown in Fig 9. Specifically, the vaccine produced elevated B- cell isotopes, which led to the development of memory cells. It was also shown by analyzing the robust cytokine and interleukin responses that the vaccine construct induces high levels of IFN-γ and IL-2.

The C-ImmSim data illustrates the consistent rise in immunoglobulin levels after vaccine delivery and significantly enhances the primary immune response (i.e., IgM, IgG1, IgG2, IgG1 + IgG2, and IgG + IgM) as shown in Fig 9 during the secondary and tertiary immunological responses. The antigen level also decreased during the secondary and tertiary immune responses. Additionally, the increased and sustained B-cell population densities and T-cell shown unequivocally the effectiveness of the vaccine design. The concentration of Th1 was observed to increase with each dose. Additionally, a greater density of macrophage and dendritic cells indicates that antigen-presenting cells effectively process and distribute antigen to CD4+ and CD8^+^ cells.

### 10. Molecular docking analysis

**Fig 10:**
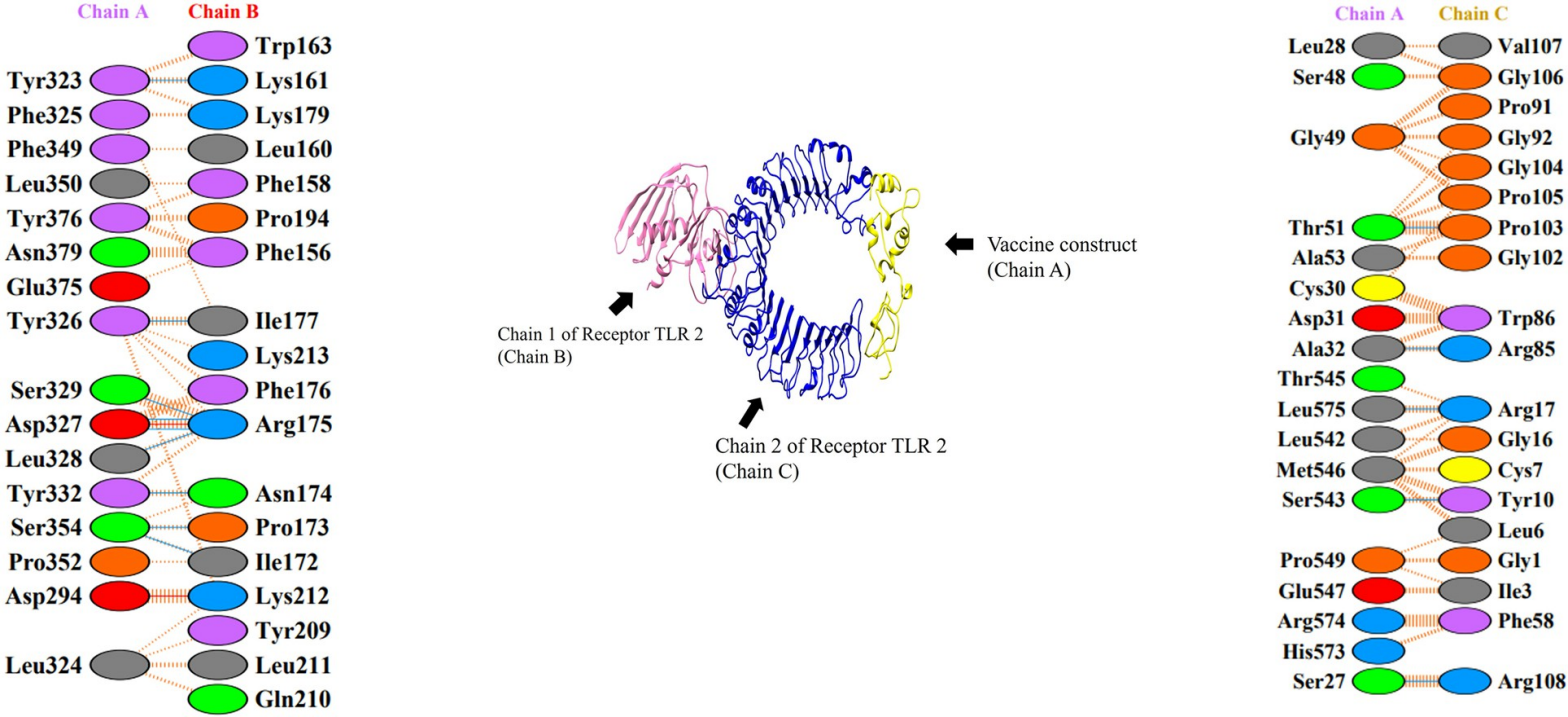
The docked complex of vaccine protein and TLR-2 receptor. Here chain 1 is represented in light blue color. chain 2 is represented in dark blue, and the vaccine protein is in yellow.

**Fig 11:**
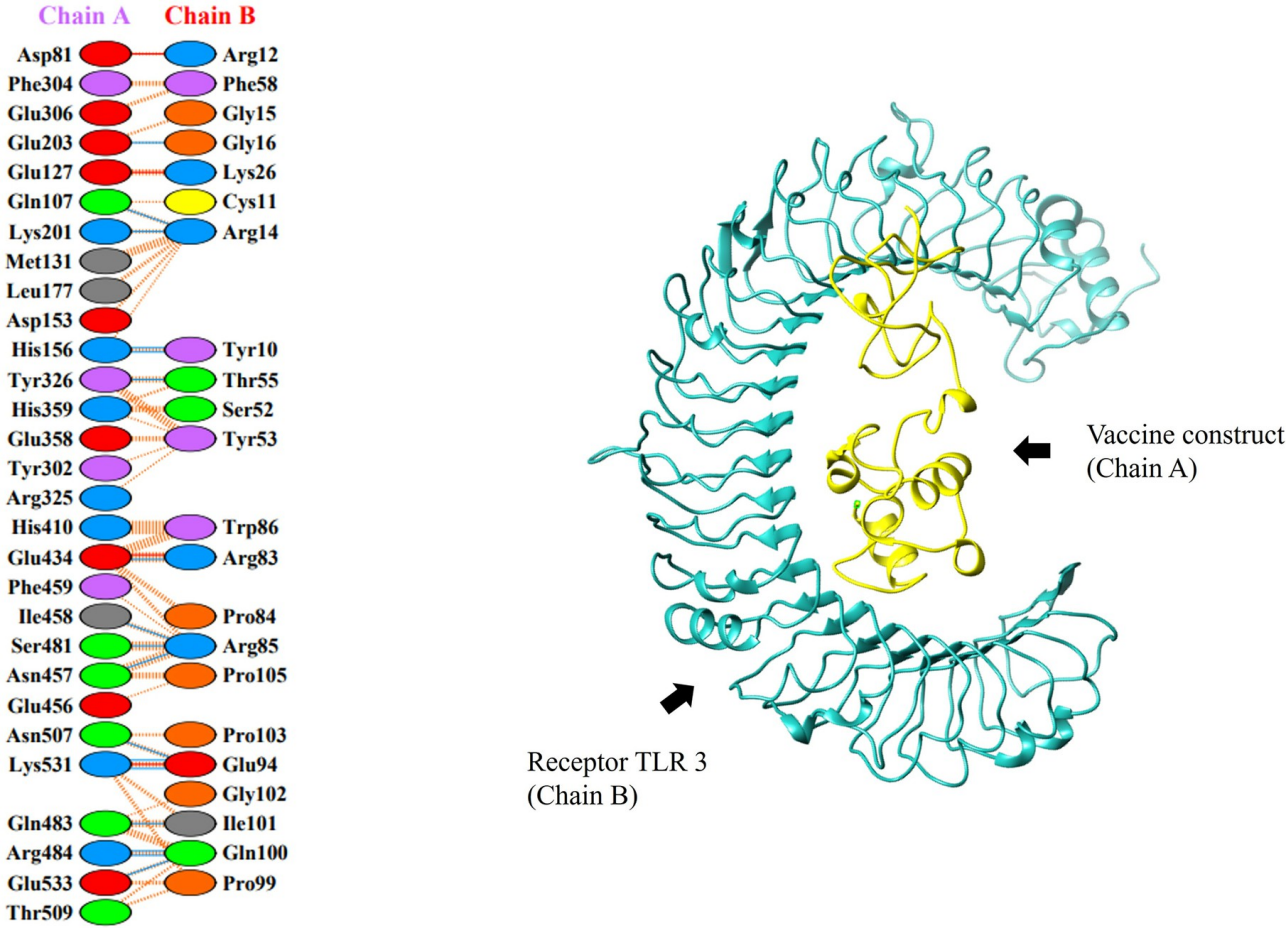
The docked complex of vaccine protein and TLR-3 receptor. Here the receptor is represented in cyan color whereas the vaccine protein is in yellow color.

Docking was done to check the vaccine binding affinity with receptor molecules. Molecular docking was done by online software ClusPro 2.0 [37] between the immune receptor, TLR2, TLR3 and vaccine construct. We use TLRs as our receptor because TLRs serve as a pathogen detection and play a crucial in innate immunity with each vaccine-TLR docking, ClusPro generated thirty clustered docked complexes in total. We used the cluster with the lowest binding energy. The lowest binding energy was -782.4 and -814.1 for TLR2 and TLR3 respectively as shown in Tables 4 and 5. The complex binding ability was best when the binding energy was lowest. Figs 10 and 11 depict the protein-protein interaction between the receptors and vaccine and the dock complexes for TLR2 and TLR3 respectively.

**Table 4:** Molecular docking results of MEV construct with receptor TLR 3.

Cluster	Members	Representative	Weighted score
0	80	Center Lowest energy	-763.0 -891.4
1	74	Center Lowest energy	-776.4 -872.2
2	73	Center Lowest energy	-813.7 -935.3
3	65	Center Lowest energy	-882.0 -882.0
4	55	Center Lowest energy	-741.2 -856.4
5	36	Center Lowest energy	-821.3 -898.8
6	35	Center Lowest energy	-734.4 -819.8
7	27	Center Lowest energy	-853.5 -853.5
8	27	Center Lowest energy	-698.8 -814.0
9	25	Center Lowest energy	-765.5 -822.6
10	25	Center Lowest energy	-841.6 -814.06

**Table 5:** Molecular docking results of MEV construct with receptor TLR 2.

Cluster	Members	Representative	Weighted score
0	82	Center Lowest energy	-781.0 -982.9
1	55	Center Lowest energy	-916.1 -984.3
2	50	Center Lowest energy	-833.7 -963.2
3	43	Center Lowest energy	-768.7 -790.8
4	42	Center Lowest energy	-734.5 -805.1
5	37	Center Lowest energy	-724.6 -797.8
6	35	Center Lowest energy	-747.4 -906.1
7	33	Center Lowest energy	-747.4 -940.1
8	29	Center Lowest energy	-764.4 -782.4
9	29	Center Lowest energy	-860.6 -901.2
10	27	Center Lowest energy	-702.6 -816.0

### 11. Molecular docking of the epitopes binding to HLA analysis

**Fig 12:**
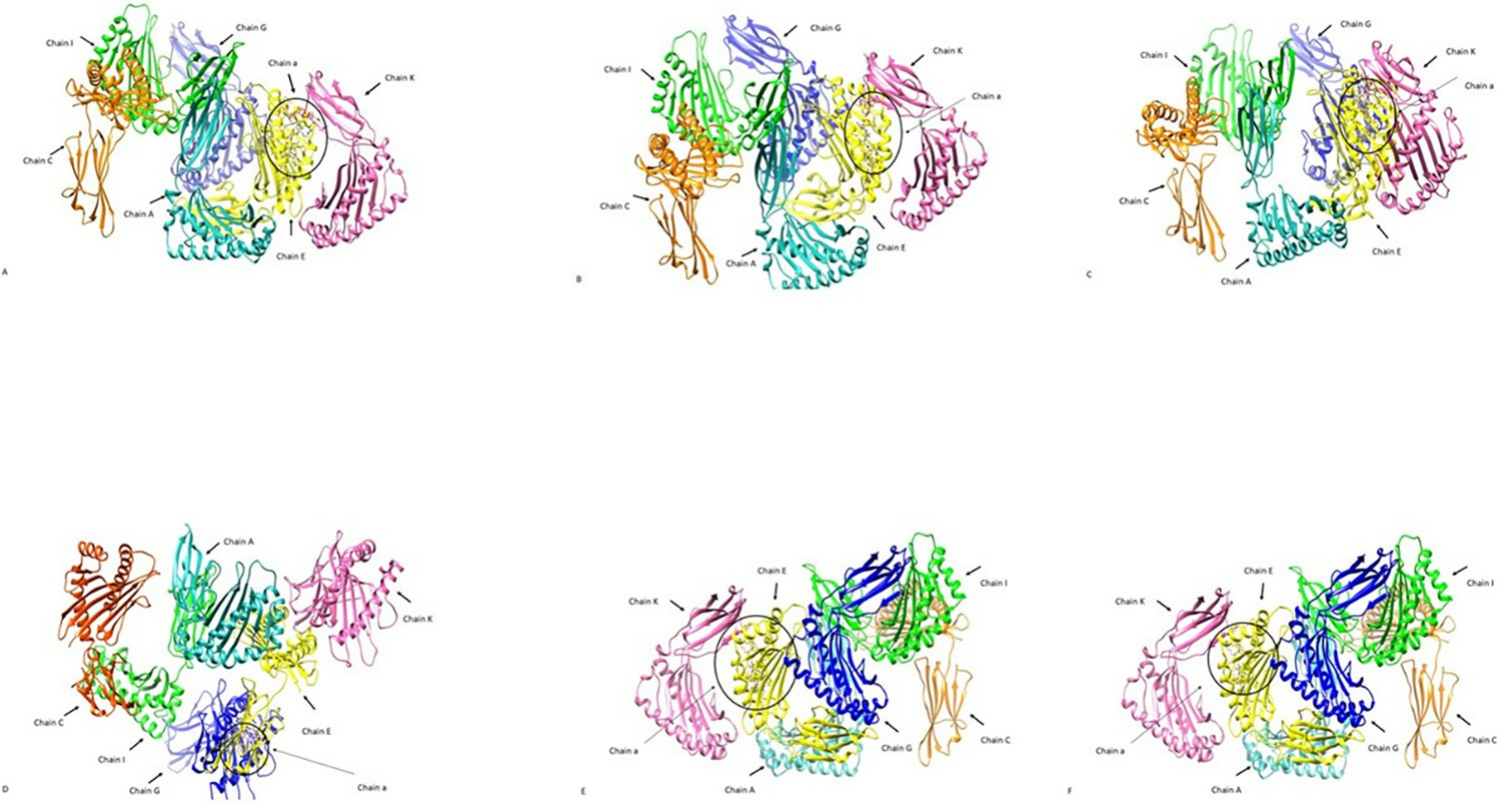
The docking interaction of six epitopes A-F with receptor HLA-A1 allele labelled as “a”. Chains of HLA-A1 alleles are chain A light sea green, chain C in orange, chain I in green color, chain G in blue, chain E in yellow and chain K in pink color while the incircle region is chain “a” of receptor HLA-A1.

Six epitopes were chosen and docked against the matching HLA-A1 allele. The **Fig 12** depicts the docking interaction of six epitopes that are labeled as A,B,C,D,E,F with receptor that is HLA A-1 alleles. According to the docking result, every epitope interacted well with the HLA-A1 allele. The binding energies of selected models of epitopes with HLA-A1 alleles are -1116.7, 735.9, -1004.0, -697.1, -881.0, -750.8 respectively. Human Leukocyte Antigen (HLA) alleles are essential for molecular docking, especially in immunology and vaccine production. Because they provide T-cells with peptides (antigenic pieces) that facilitate immunological identification, HLA molecules are essential to the immune system. They are particularly significant in docking investigations because of several reasons such as: T-cells are presented with peptides by HLA molecules and trigger an immunological response. The ability of a peptide to bind to a particular HLA allele is predicted by using docking. The peptide’s likelihood of being recognized by the immune system as "foreign" is determined by its binding affinity. In order to ensure greater efficacy for people or communities, docking studies aid in the formulation of treatments or vaccinations customized to particular HLA types. Finding peptides that bind to common HLA alleles with high strength can help direct the creation of vaccines based on epitopes. Docking forecasts which pathogen components are most likely to elicit a strong immune response.

### 12. Normal mode analysis

**Fig 13:**
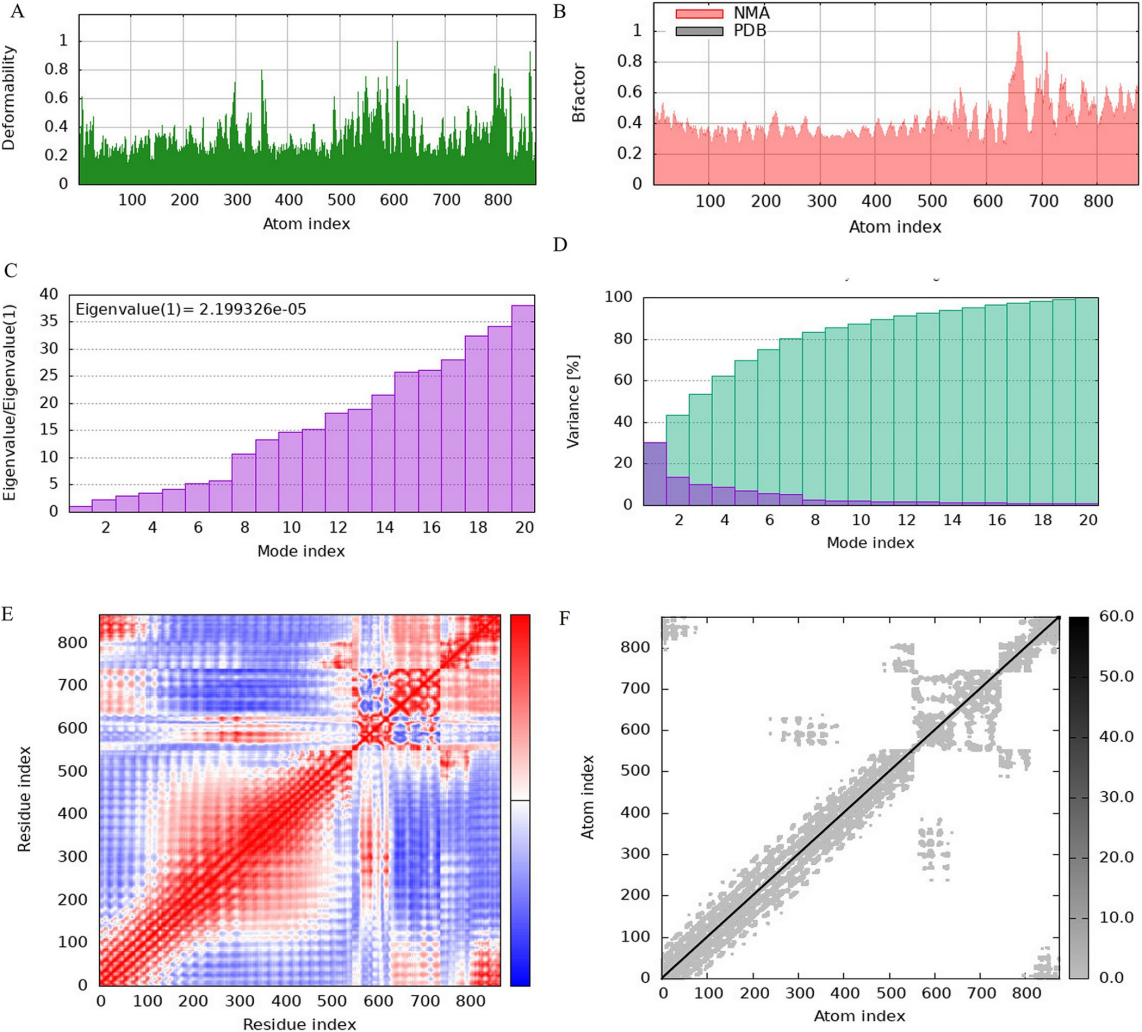
Normal mode analysis for TLR 2**(A)** Deformability **(B) B-**factor **(C)** Eigenvalue **(D)** Variance **(E)** Covariance **(F)** Elastic network model.

**Fig 14:**
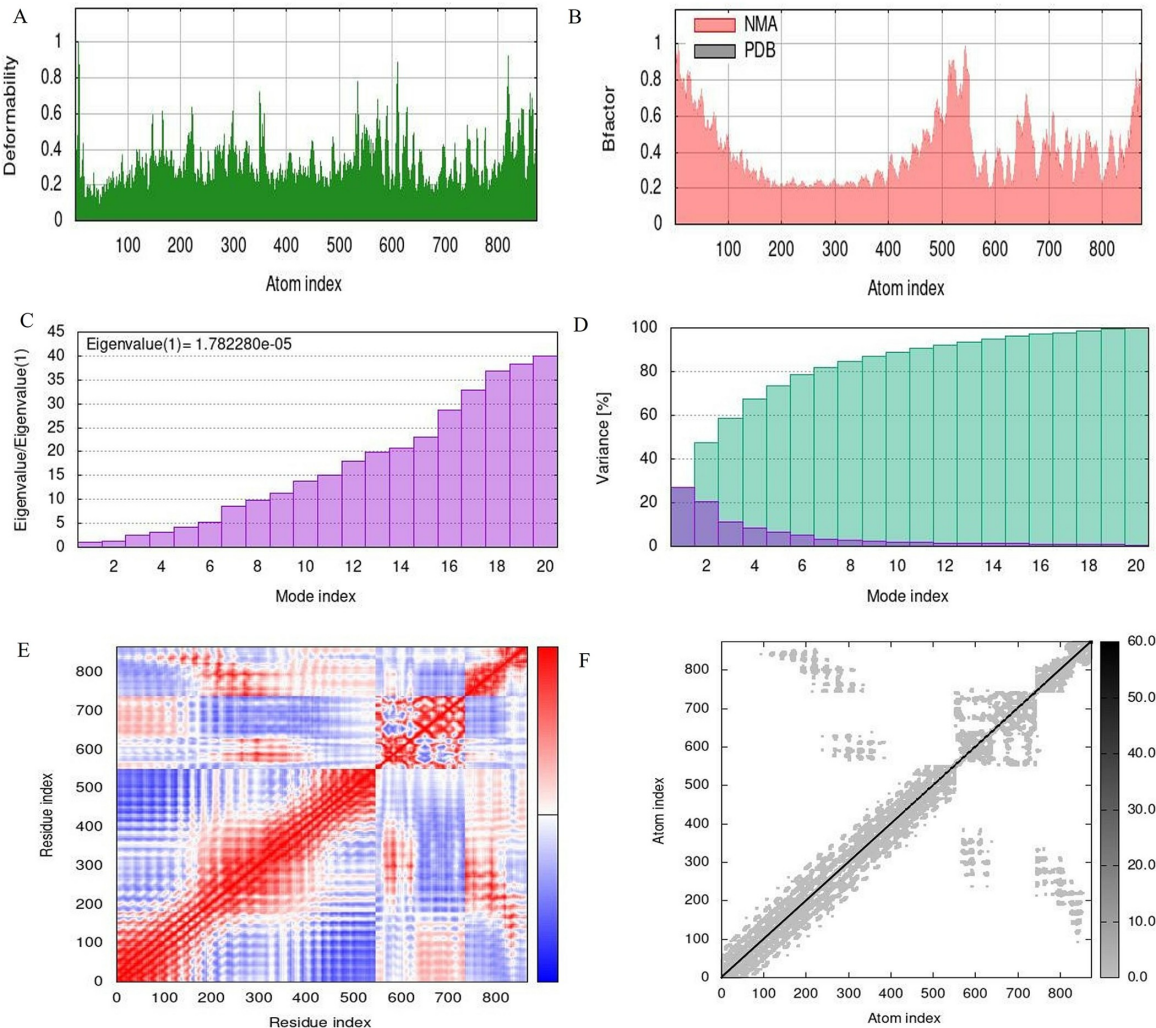
Normal mode analysis for TLR 3**(A)** Deformability (B) **B-**factor **(C)** Eigenvalue (D) Variance (E) Covariance (F) Elastic network model.

For a thorough examination of the mobility and stability of proteins, normal mode analysis (NMA) was done using IMODE server [38]. One of the common methods for illustrating the large-scale mobility and stability of proteins is normal mode analysis. Here B- factor authenticates the normal mode analysis as shown in Figs 13A and 14A for TLR2 and TLR3 respectively. The center of the polypeptide molecule and the beginning of the TLR molecules are the regions with the most capability to deform, according to the deformability index graph as shown in Figs 13B and 14B. Eigenvalues represent stiffness (Figs 13C and 14C). It is simpler to distort the complex with a lower eigenvalues value. Variance and eigenvalue have an antagonistic relationship. As shown in the Figs 13D and 14D, light blue indicates cumulative variances and purple represents individual variances, respectively. The covariance chart as shown in (Figs 13E and 14E) displays motions that are anti-correlated (blue), uncorrelated (white), or correlated (red). The stiffer elastic network is denoted by darker gray patches.

Concurrently, a decrease in the average polar solvation relative to the initial structure revealed an increase in the complex’s compactness, which validates our results on the complex’s Rg. Additionally, using server iMODS to enumerate normal mode analysis (NMA), the stiffness in the mobility and deformability in the complex’s residues were examined. Less mobility in the residues was shown by the complex’s deformability plot, and the examined NMA also showed less deviation from the complex’s B-factor. The complex structure’s calculated eigenvalue increased steadily in each mode (Fig 13C). The individual variance of each successive mode decreased moderately, according to the variance plot analysis (Fig 13D).

## Discussion

HCV is a blood borne pathogen that affects millions of individuals worldwide. In 70% of the cases, HCV causes persistent infections by evading host immune response. Several risk factors such as gender, age, and ethnic group, influence chronic HCV infection [39] and 3% of the population get infected with HCV infection globally. DAA successfully treats infection but does not induce immunological memory responses [40]. With seven genotypes and other subtypes that are sporadically distributed throughout the world, HCV demonstrates a high degree of genetic heterogeneity. To guarantee efficacy across genotypes, a potential vaccine would have to target conserved areas of the virus, such as the nonstructural proteins or conserved epitopes in the envelope proteins. Epitope based vaccines that target conserved HCV antigens may provide widespread protection by boosting cellular immunity, potentially overcoming strain and regional diversity, according to preliminary studies and clinical trials. The multi epitope peptide-based vaccines using immunoinformatic approach appeared as progressive technique over conventional methods in terms of effectivity and efficacy. This research work is based on an innovative idea of vaccine designing and helps to prevent the spread of HCV infection. Using computational methods to anticipate antibody epitopes is a crucial step in the vaccine designing [41]. NS5B, a crucial RNA-dependent RNA polymerase (RdRp) of HCV, is necessary for the replication of the viral genome. It is a prime target for the development of antiviral drugs due to its distinct structure and catalytic activity. It has great significance as a universal antiviral target and is further highlighted by the fact that it is conserved among HCV genotypes.

Immunoinformatic techniques represent a significant advancement in the development of multi epitope vaccines. Essential steps for developing multi-epitope vaccine include identifying B-cell epitopes and T-cell epitopes. While keeping the defensive strategy and therapeutic potency of the generated vaccine candidate, the construction of a multiepitope vaccine construct against HCV has produced encouraging findings. Previous research also suggested that identifying potential epitopes is thought to be essential for creating multi epitope peptide based vaccines [42]. T- cell and B-cell epitope prediction is a necessary and crucial step in the construction of multi-epitope peptide vaccines due to the reasons that T- cells recognize pathogen-derived antigens and perform direct effector actions such as cytotoxicity and assist B-cells in controlling the maturation and development of antibody responses [42,43].

The rational design of multi-epitope vaccines requires the identification of immunogenic epitopes derived from pathogen antigens that can be digested and presented by dendritic cells (DCs). The desired shortlisted epitopes present a promising approach to accelerate vaccine development when paired with the *In-Silico tools*. The potential efficacy of the vaccine is highlighted by the strong immune responses, advantageous physiochemical properties, and high population coverage that was observed in simulations [44].

This study describes a process for designing a vaccine that can provide defense against HCV. We designed candidates that maximize the best covered epitopes for each NS5B protein version of the Hepatitis C virus [45,46]. To analyze sequence conservation, MSA and positional diversity of sequences was performed using the CLC main workbench. ABCpred was used to predict the B-cell epitope and the IEDB to identify the most effective epitopes for the T-cell epitopes. MHC pred [32,47] can be used to determine binding affinity. Using the web server VaxiJen [48], the epitopes antigenicity was assessed. To assess certain allergenicity, the antigenic epitopes with threshold 0.6 were subsequently entered into the web application AllerTop [44]. Using Innovajen, epitopes free of allergens were tested for solubility; those that were soluble were then tested for toxicity using toxin-pred [49–51].

It is believed that immunodominant multi-epitope based vaccine are a viable therapy option for pathogenic and viral infections. To improve the vaccine’s immunogenicity and efficacy, an adjuvant was added throughout the design process. Adjuvants enhance the immunologic qualities of vaccine structures, making them crucial parts of multi-epitope vaccines [52]. Adjuvant (β-defensin) was added to vaccines at the N-terminus to boost their antigenicity because it has been shown to improve memory T cells, monocytes, and immature dendritic cells chemotactic activity. It might also enhance the delivery of the vaccines and shield them from deterioration [53]. Beta-defensins are especially helpful in vaccines that target difficult infections like HCV, HIV, or malignancies where robust and long-lasting protection is needed because of their capacity to enhance humoral and cellular immune responses.

Adjuvant human β-defensin was added to vaccines at the N-terminus to boost their antigenicity because it has been shown to improve memory T cells, monocytes, and immature dendritic cells chemotactic activity. It might also enhance the delivery of the vaccines and shield them from deterioration [53]. We used Beta defensin as an adjuvant linked to the epitopes utilizing the EAAAK linkers. GPGPG linkers work in conjunction with additional epitopes [54]. The function of the linker is to guarantee that every epitope can initiate an immune response on its own and to inhibit the emergence of other epitopes that could potentially disrupt the immunological response that the original epitope triggered [34].The stability of the anticipated model was demonstrated by the Ramachandran plot validation. When sulfur atoms create cysteine pairs in protein structures, the disulfide bonds are established covalently. It promotes the stability of protein structures [55]. Disulfide engineering was done to get rid of any potential instability and improve the vaccine’s overall stability [24]. One essential technique for producing proteins is recombinant protein expression, which is achieved with bacteria and other host organisms. Codon optimization, a crucial stage in the production of recombinant proteins, involves designing a protein’s coding sequence by synonymous replacement to maximize its expression level. Codon optimization is done to enable effective protein expression the ultimate vaccine design was inserted into the expression vector using snapgene [28]. C-Immsim a computational immune simulation tool was employed to evaluate the vaccine’s ability to mount an immune response and predict potential immune responses. After that, the completed construct was docked against TLR3 and TLR2 by using Cluspro 2.0 tool to examine sufficient binding to elicit an immunological response, TLR3 activates antiviral defenses during infection and aids in the detection of infectious dead cells [56]. Toll-like receptors (TLRs) are essential class of macromolecules in the immune system. These days, everyone understands how crucial TLRs are in connecting the innate and adaptive immune systems. Pathogen-associated molecular patterns (PAMPs), microbial/microbe-associated molecular patterns (MAMPs), damage/danger-associated molecular patterns (DAMPs), and xenobiotic-associated molecular patterns (XAMPs) bind to the TLRs and activate them [57].TLR stimulation induces the production of proinflammatory cytokines and chemokines [58].

By offering a computationally driven method to HCV vaccine design, the manuscript makes a substantial contribution. It highlights how effective immunoinformatics techniques are at locating and enhancing antigenic epitopes, opening the door to accurate and effective vaccine production. The findings of the authors demonstrate how computational research might expedite vaccine development by lowering the expenses and duration of conventional experimental techniques while maintaining high safety and specificity. But there are still issues to be resolved, such as ensuring sufficient coverage of every genotype and resolving regional differences in HCV prevalence and access to care, which may affect vaccine efficacy.

This work strengthens the significance of computational approaches in contemporary biomedical research by advancing our understanding of HCV vaccine development and showcasing their wider promise in the fight against other difficult infections.

## Conclusion

This study marks a significant advancement in the development of multi-epitope based vaccine to prevent HCV infection. The study used NS5B of HCV to predict potential epitopes and design a vaccine based on epitopes, which is a novel approach to date. Furthermore, the accuracy and breadth of this investigation were greatly improved by the construct effective docking with the antigenic receptor. This study also highlights that computational vaccinology plays a pivotal role in expediting the development of vaccines by providing a clear and efficient method. Key challenges posed by HCV are addressed by the multi-epitope vaccine design, which lays the groundwork for additional experimental validation and clinical investigation. Public health depends on the development of effective and preventive measures such as vaccine, since the global burden of HCV is still significant.
